# Evolution of Immunotherapy for Ovarian Cancer from a Bird’s-Eye Perspective: A Text-Mining Analysis of Publication Trends and Topics

**DOI:** 10.3389/fonc.2022.795129

**Published:** 2022-02-24

**Authors:** Guangyi Jiang, Junjie Hong, Feng Shao, Qiang Wen, Feng Cheng, Tunan Yu, Jianqing Zhu

**Affiliations:** ^1^ Department of Gynecological Oncology, Cancer Hospital of University of Chinese Academy of Sciences (Zhejiang Cancer Hospital), Hangzhou, China; ^2^ Department of General Surgery, Sir Run Run Shaw Hospital, College of Medicine, Zhejiang University, Hangzhou, China

**Keywords:** ovarian cancer, immunotherapy, bibliometric, subtopic trends, text-mining analysis

## Abstract

**Objectives:**

Ovarian tumors are among the most prominent gynecological malignancies and have a poor prognosis. Immunotherapy has undergone incredible progress in the past two decades. Our study aimed to use a bibliometric approach to identify research trends in ovarian cancer immunotherapy.

**Methods:**

Literature on this topic published from 2000–2020 was retrieved from the Web of Science Core Citation database and analyzed using the bibliometric analysis software VOSviewer and CiteSpace.

**Results:**

A total of 1729 articles on ovarian cancer immunotherapy published from January 2000 to December 2020 were identified. The number of published articles increased each year, from 40 in 2000 to 209 in 2020. These publications were from 61 countries, and the USA showed a dominant position in publication output, total citations, and average number of citations per paper. Co-citation networks revealed 14 subtopics. ‘PD-L1 expression,’ ‘tumor reactive til,’ and ‘parp inhibitor’ are the current potential subtopics. Furthermore, we determined research trends according to the timeline analysis.

**Conclusion:**

Our study exhaustively describes the development and summarizes the research trends of ovarian cancer immunotherapy over the past 20 years.

## 1 Introduction

Ovarian cancer (OC) is the leading cause of gynecological malignant tumors. Due to vague clinical symptoms and lack of guidelines for OC screening, > 70% of patients present with late-stage (III–IV) OC at the first diagnosis and have a poor prognosis (1, 2). The five-year survival rate is approximately 45–47%, primarily due to chemoresistance and tumor relapse (3). Cytoreductive surgery and adjuvant chemotherapy, which recommend a platinum/taxel combination as the first-line treatment, have become the standard therapy for OC. However, about 50–70% of patients will experience chemoresistance that hinders therapeutic efficacy (4). Research on the pathological mechanisms of OC shows that homologous recombination deficiency (HRD), which relates to cancer susceptibility, caused by key gene mutations, such as BRCA1/2, occurs in 41–50% of ovarian carcinomas (5). Poly (ADP-ribose) polymerase (PARP) inhibitors cause synthetic lethality by compromising two pathways of DNA repair in HRD patients (6). Several clinical trials showed that PARP inhibitors are associated with progression-free survival regardless of BRCA mutation status (7–9). However, PARP inhibitor resistance in BRCA1/2-mutated tumors means that the effect of PARP inhibitors is insufficient (10, 11). The efficacy of established therapies has plateaued (12, 13), and this underscores the need for new treatment strategies and paradigms for patients with OC.

Immunotherapy, which involves the activation of endogenous immune response to eliminate tumor cells, appears to be the new frontier of anticancer treatment (14). For years, cancer immunology research has achieved incredible milestones. Currently, multiple immunotherapeutic modalities are being developed and tested in clinical trials (15). Immunotherapeutic approaches for OC, such as antibody-based therapies, immune checkpoint blockade, cancer vaccines, and chimeric antigen receptor-modified T cells, have demonstrated preclinical success and have entered clinical testing.

By reviewing the research literature on OC immunotherapy, we can observe the phenomenon of literature proliferation. The research focus in this field changes according to the efficacies of clinical treatments. Some of these topics attract steady interest while others may be passing or recurring trends. Therefore, identification of current research hotspots and future trends is helpful for the optimal development of the field. Bibliometric analysis that relies on an artificial intelligence-based algorithm is advantageous for text-mining of information extracted from research literature (16). Bibliometric analysis is superior to traditional review in terms of information data processing and visualization by utilizing mathematical and statistical procedures to recognize and organize knowledge structure (17). It ensures better review of research development in a particular field, as well as current research practices.

The main aim of our study was to review the development of and delineate the main trends of published literature about OC immunotherapy. In particular, we focused on shifts in current topics and geographic trends.

## 2 Materials and Methods

### 2.1 Data Collection

**Figure 1:**
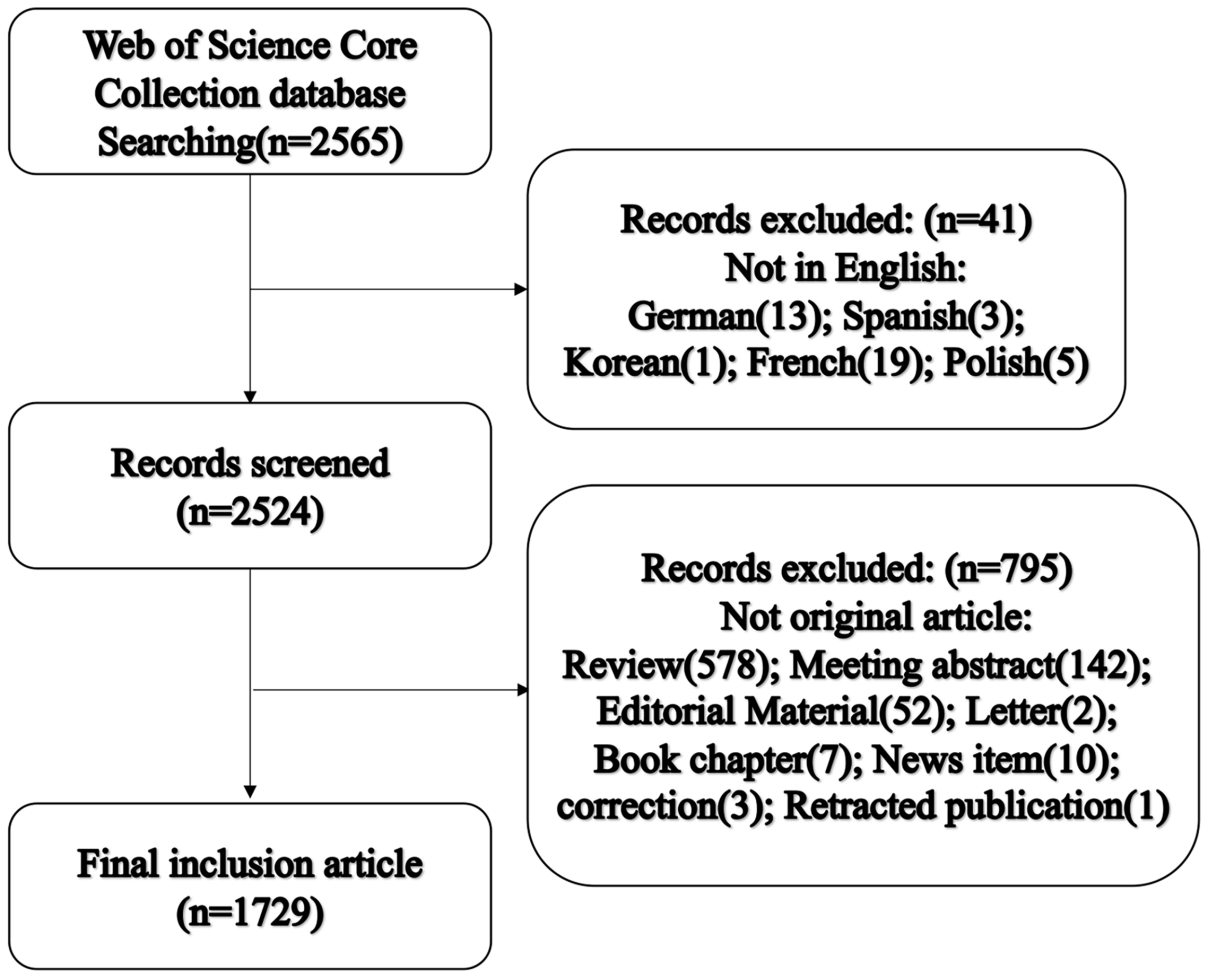
Flow chart of literature inclusion and exclusion.

The Web of Science Core Citation (WOSCC) database was searched for literature on OC immunotherapy from January 2000 to December 2020. The main definition keywords utilized were OC- (Strategy A) and immunotherapy-related (Strategy B) Terms (**Supplemental Table 1**). Finally, a Boolean algorithm as “A AND B” (Strategy C) was utilized to ensure that all articles retrieved were in the field of OC immunotherapy. Only original research articles were included in our analysis, and the language was restricted to English. We exported the following details with the record content of “Full Record and Cited Reference,” which comprised year of publication, authors, title, abstract, keywords, journal, country, and references. The flow diagram is shown in **Figure 1**. This study was designed in line with the PRISMA guidelines.

### 2.2 Analysis Methods and Visualization

Data processing and result visualization were done using bibliometrics software CiteSpace (5.3.R4) (Chaomei Chen, Drexel University, USA) and VOSviewer (1.6.6).

CiteSpace, a bibliometric program, was utilized to extract the keywords with period bursts (18). The clustering function was used to identify subject foundation and current hotspots. The clustering procedure was as follows: First, a co-citation network for immunotherapy of OC literature was constructed and simplified with the strategy of “top 50 cited publications per year.” Second, influential articles were identified based on citations to seed clusters. Third, articles co-cited with the seeding article of a cluster were assigned to the cluster. The label of each cluster was summarized mainly based on keywords from log-likelihood algorithm. The timeline map was built based on clustering results. Citation maps from CiteSpace were visualized and exported as a PNG file.

VOSviewer (https://www.vosviewer.com) is a program operated by the Centre for Science and Technology Studies at Leiden University for constructing and visualizing bibliometric networks (19). VOSviewer can construct and visualize the keyword co-occurrence network. Compared with CiteSpace, VOSviewer software has advantages in drawing images and making the network more intuitive through the time axis, which can be more helpful in understanding the hot topics and current trends (20). Citation analysis was used to demonstrate the co-cited countries and organizations based on bibliographic data. The visualization of the related citation map was produced correspondingly and exported as a PNG file.

### 2.3 Patient and Public Involvement

No patients were involved.

## 3 Results

**Figure 2:**
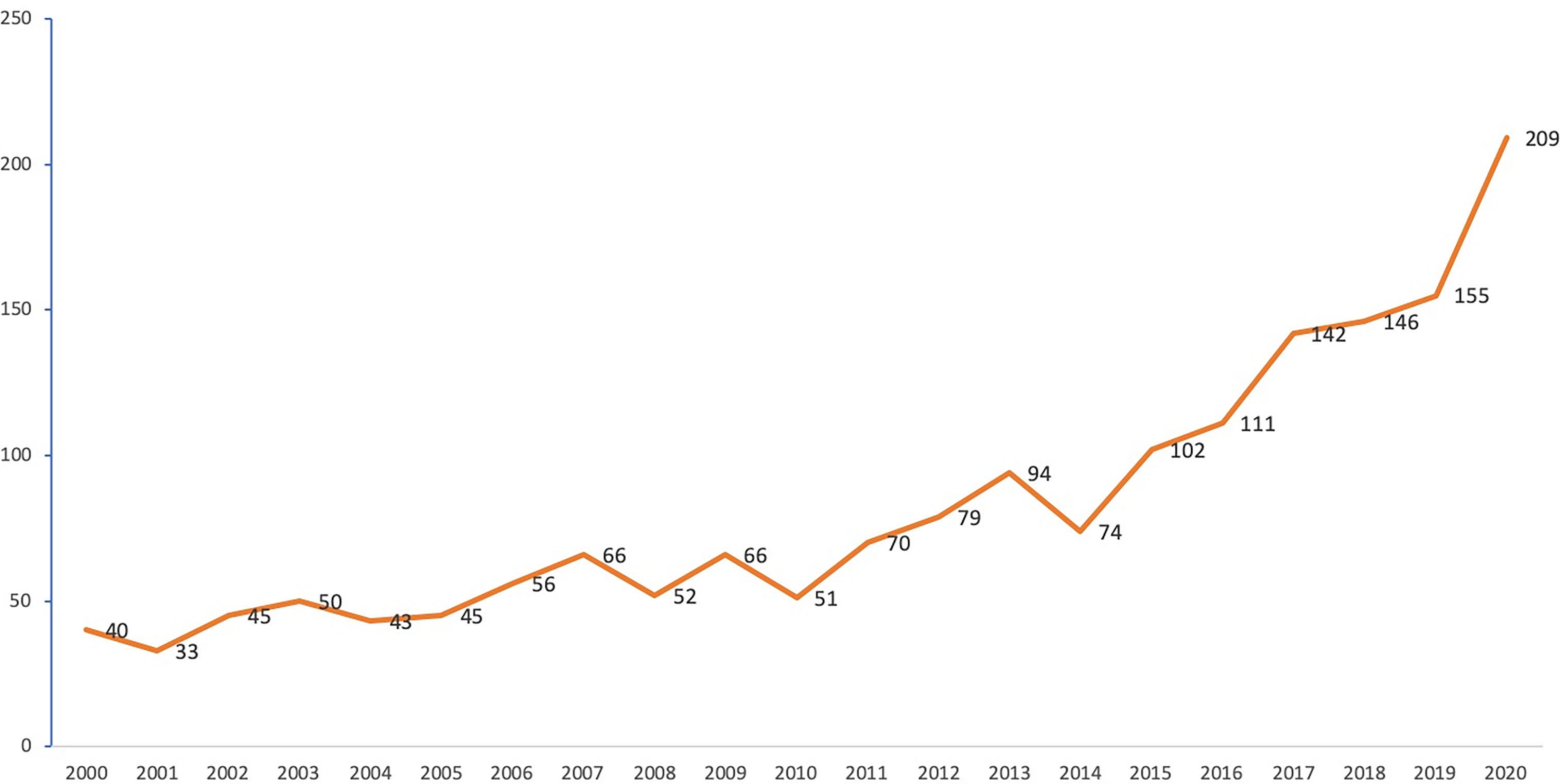
Annual publication number in the field of ovarian cancer immunotherapy.

According to the WOSCC database search results, a total of 1,729 published articles were included in our analysis. The annual publications from 2000 to 2020 are listed in **Figure 2**. The number of annual publications varied from 40 in 2000 to 209 in 2020.

### 3.1 Geographic Trends

**Figure 3:**
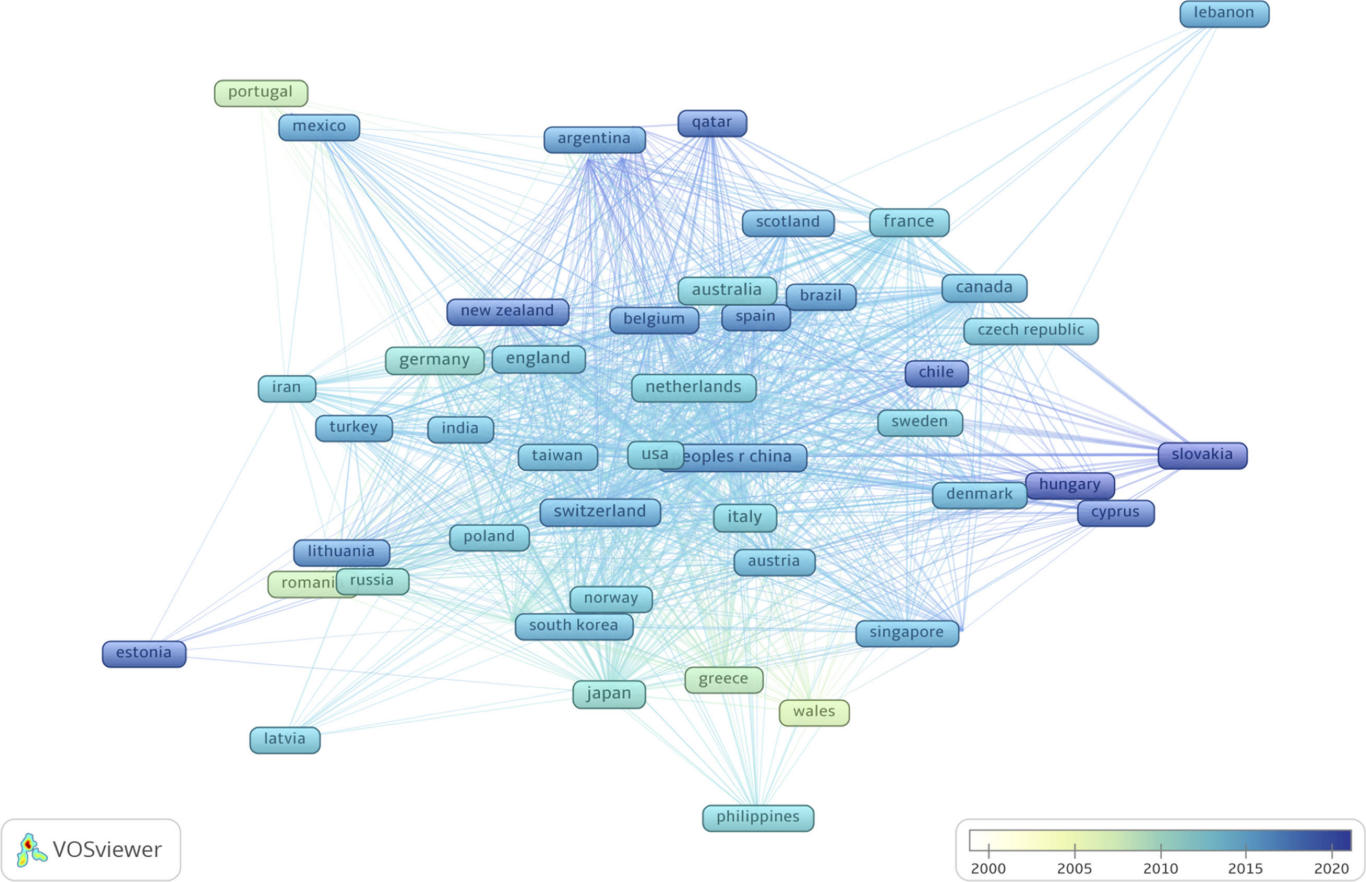
Co-citation network of countries based on VOSviewer. The color of node and line represents different years, colors vary from white to deep blue as time goes from 2000 to 2020 and the links indicate the co-occurrence relationships.

A total of 56 countries have published studies on OC immunotherapy. The network of countries produced by VOSviewer is shown in **Figure 3**; the node size represents the total number of publications from each country and the lines between nodes represent citations. The United States of America (USA) was the most productive country with 798 published articles, followed by China (n=300), Germany (n=148), Japan (n=125), Italy (n=110), England (n=110), Canada (n=90), France (n=80), the Netherlands (n=64), and Switzerland (n=49). Articles published in the USA had the highest total and average number of citations. China had the second largest number of publications and the third highest number of citations overall but the lowest average number of citations per study. Moreover, we observed that countries such as Portugal, Romania, Greece, and Wales conducted research in the early 2000s, while Qatar, Chile, and Slovakia have only begun conducting OC studies in recent years. Globally, a total of 2,232 institutions published research on OC immunotherapy independently or co-operatively. The top 5 institutes were University of Pennsylvania (U.S., 104 papers, 6.2%), Memorial Sloan Kettering Cancer Center (U.S., 70 papers, 4.0%), National Cancer Institute (U.S., 65 papers, 3.8%), The University of Texas MD Anderson Cancer Center (U.S., 59 papers, 3.4%), and Roswell Park Cancer Institute (U.S., 58 papers, 3.4%).

### 3.2 Subtopic Trends

**Figure 4:**
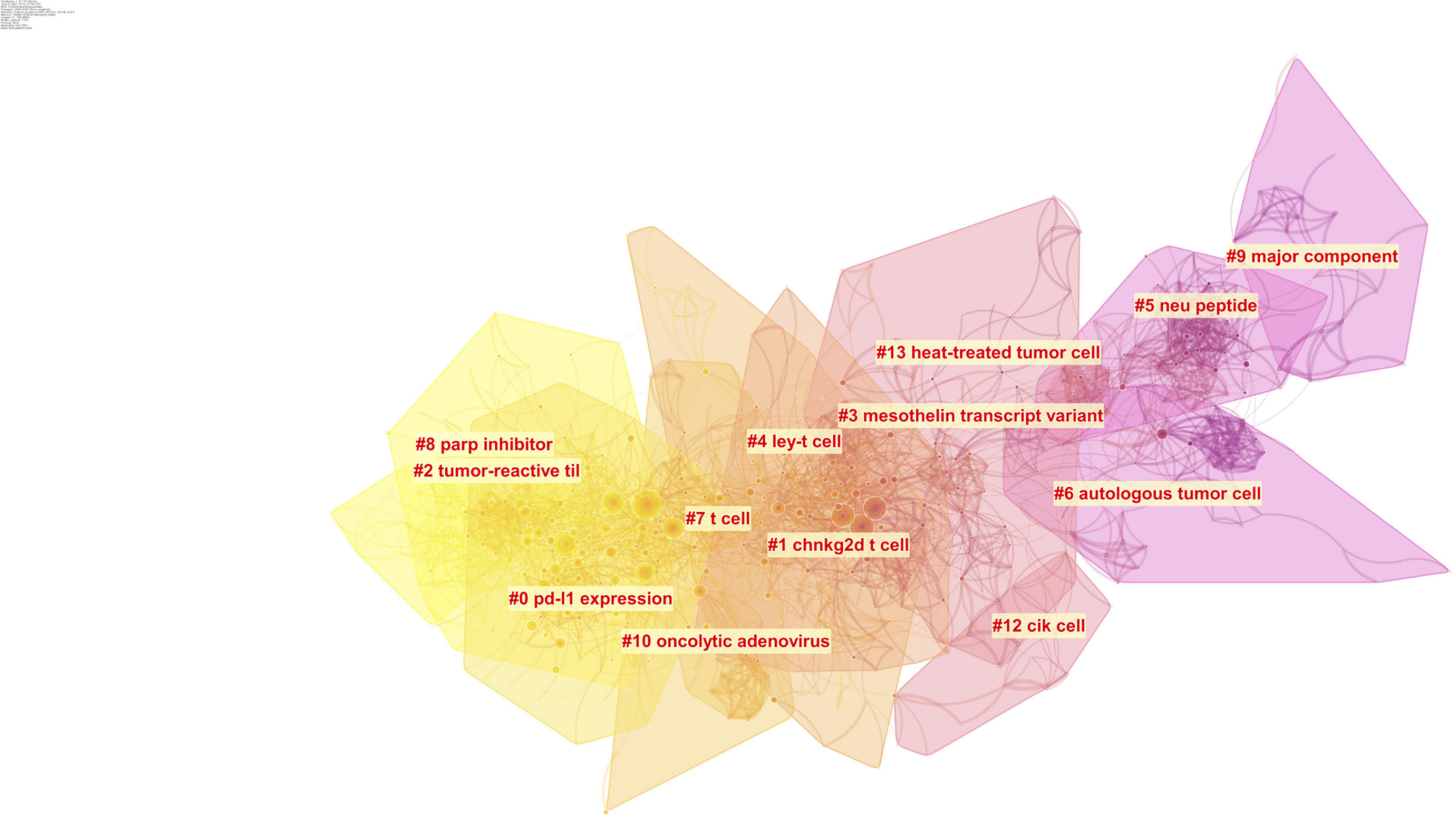
Clustering of the co-citation network of the references. The color represents different cluster, colors vary from yellow to pink represent the prevalence time, The closer yellow represents the closer cluster prevalence time to the present, the closer pink the opposite.

Intellectual base and research fronts are two typical indicators of a research program in a research field. The intellectual base is the combination of scholarly works that have been cited by the corresponding research field, whereas research fronts are the works that arise from the intellectual base. The reference of all the documents in a research field constitutes the foundation of that field. Through citation analysis, we can understand the intellectual base and developmental process of the field and further explore research hot spots by extracting keywords. A total of 46,972 references were cited by the included articles, and 14 major subtopics pertaining to OC immunotherapy were identified using a clustering function (**Figure 4**). The network has a modularity of 0.7261, which is considered very high. This suggests that the specialties are clearly defined in terms of co-citation clusters. The average silhouette score of 0.3262 is relatively low. This is mainly because only the major clusters can be visualized, and some small clusters with low silhouette scores did not appear. The major clusters that we will focus on in the review have a sufficiently high silhouette score. In other words, one would consider these clusters to be specialized and with high level of homogeneity. The results of the clustering are credible.

**Figure 5:**
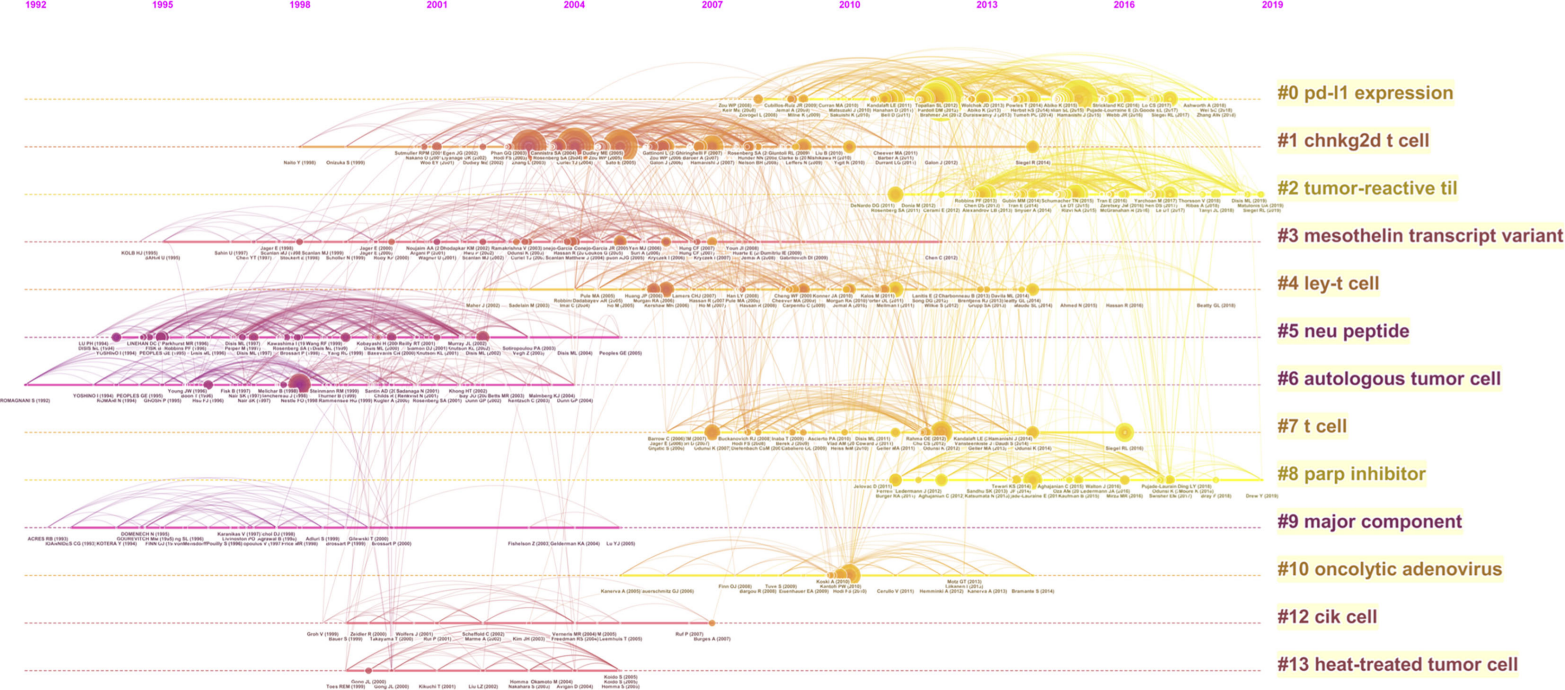
A timeline visualization of the 14 clusters.

Timeline view is a time axis that explores a research field in a specific period, and it provides a very intuitive and accurate reference for us to understand the evolution pathway of each subtopic. Throughout the past two decades, several subtopics within the field of OC immunotherapy were popular at various times (**Figure 5**). Node sizes were determined by highly cited references that had a high impact on each subtopic. The subtopics that have been active in recent years are cluster #0 ‘PD-L1 expression,’ cluster #2 ‘tumor reactive til,’ and cluster #8 ‘parp inhibitor.’ The ‘PD-L1 ex-pression’ subtopic first occurred in 2008, and this was followed by a burst of studies until 2018. The ‘tumor reactive til’ and ‘parp inhibitor’ subtopics were prevalent from 2011 to 2020. The cluster#1 ‘chnkg2d t cell’ subtopic showed a burst of activity from 2003 to 2009, and then the research boom slowly declined.

Three bibliometric indices of centrality, burstness, and sigma were used to identify the top 5 publications of significance in the science domain (**Table 1**). These three bibliometric indices are used to evaluate the importance of the literature in this field, and the values represent the contribution value of the literature to the domain and mark a milestone in literature.

**Table 1:** Top 5 papers according to three bibliometrical indices in the field of immunotherapy of ovarian cancer.

Year	Authors	Journal	Title	Bibliometric indices		
				Centrality	Burstness	Sigma
2015	Hamanishi J et al.	Journal of Clinical Oncology	Safety and Antitumor Activity of Anti-PD-1 Antibody, Nivolumab, in Patients With Platinum-Resistant Ovarian Cancer		25.24	
2004	Curiel TJ et al.	Nature Medicine	Specific recruitment of regulatory T cells in ovarian carcinoma fosters immune privilege and predicts reduced survival	0.13	29.4	7.91
2003	Zhang L et al.	The NewEngland Journal of Medicine	Intratumoral T cells, recurrence, and survival in epithelial ovarian cancer		29.64	4.27
2005	Sato E et al.	PNAS	Intraepithelial CD8+ tumor-infiltrating lymphocytes and a high CD8+/regulatory T cell ratio are associated with favorable prognosis in ovarian cancer		27.42	6.89
1998	Nestle Fo et al.	Nature Medicine	Vaccination of melanoma patients with peptide- or tumor lysate-pulsed dendritic cells	0.16	15.98	10.12
2007	Hamanishi J et al.	PNAS	Programmed cell death 1 ligand 1 and tumor-infiltrating CD8+ T lymphocytes are prognostic factors of human ovarian cancer	0.22		9.2
2002	Dudley ME et al.	Science	Cancer Regression and Autoimmunity in Patients After Clonal Repopulation with Antitumor Lymphocytes	0.17		
2010	Hodi FS et al.	The NewEngland Journal of Medicine	Improved Survival with Ipilimumab in Patients with Metastatic Melanoma	0.13		

### 3.3 Major Specialties

In the following sections, we further analyzed the two largest clusters with significant high-impact cited references.

#### 3.3.1 Cluster #0 – PD-L1 Expression

**Figure 6:**
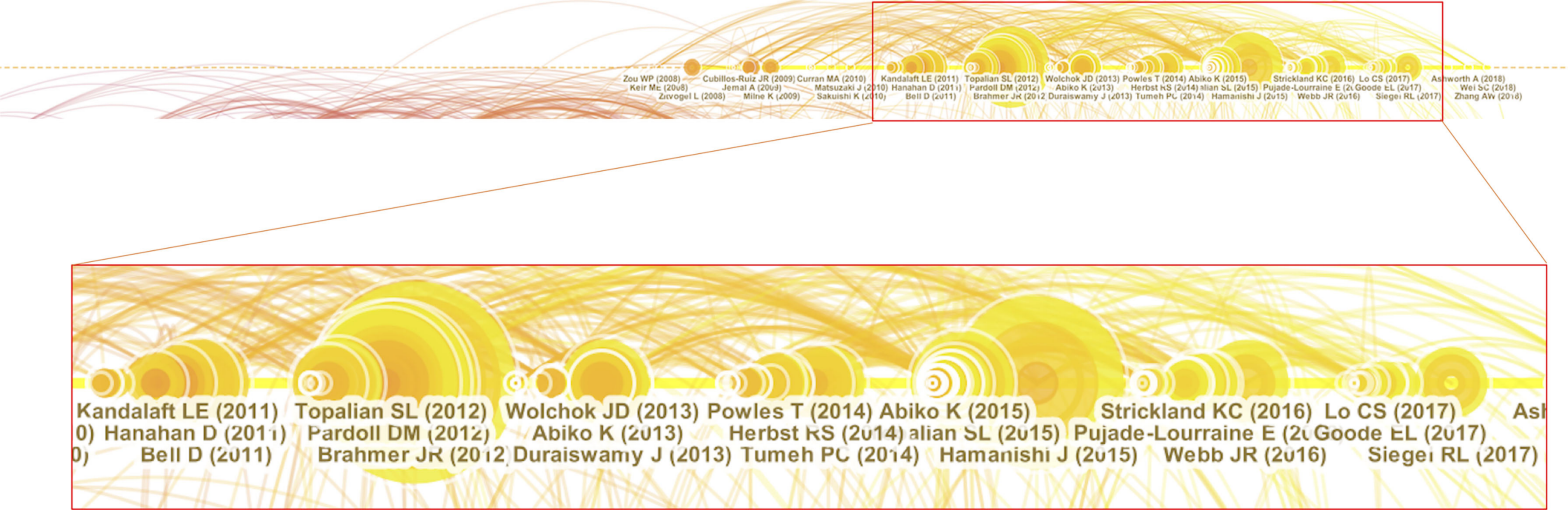
High impact members in cluster #0.

Cluster #0 was the largest cluster and contained 137 references. The silhouette score was 0.822, and the median year of all the references in the cluster was 2013. Timeline visualization revealed that the subtopic about PD-L1 expression was prevalent from 2008 to 2018, and highly cited references were published in this field annually (**Figure 6**). This period is full of high-impact contributions that had large citation numbers and periods of citation bursts.

Notably, several high-impact contributions appeared in this period. Two clinical trials were conducted to assess the safety and activity of anti-PD-1 (Topalian et al., 2012) (21) and anti-PD-L1 (Brahmer et al., 2012) (15, 22) antibodies in patients with selected advanced cancers. These two antibodies induced durable tumor regression and prolonged disease stabilization in patients with non-small-cell lung cancer, melanoma, and renal cell cancer. However, the response rate of the anti-PD-L1 antibody in patients with OC was low. Abiko et al. demonstrated that PD-L1 expression on OC cells can be induced by ascites lymphocytes and can promote peritoneal dissemination (Abiko et al., 2013) (23). Another study by Abiko et al. reported that CD8 positive T cells in OC stroma can secrete interferon-γ and upregulate PD-L1 expression on OC cells and thus promote tumor growth (Abiko et al., 2015) (24). Notably, the most cited publication was the first clinical trial of nivolumab for treating platinum-resistant OC in which it was demonstrated that the drug had a 15% overall response rate (ORR) and a 45% disease control rate. The median progression-free survival time was 3.5 months, and the median overall survival time was 20.0 months by the end of the study (Hamanishi et al., 2015) (25).

#### 3.3.2 Cluster #1 – chnkg2d T Cell

**Figure 7:**
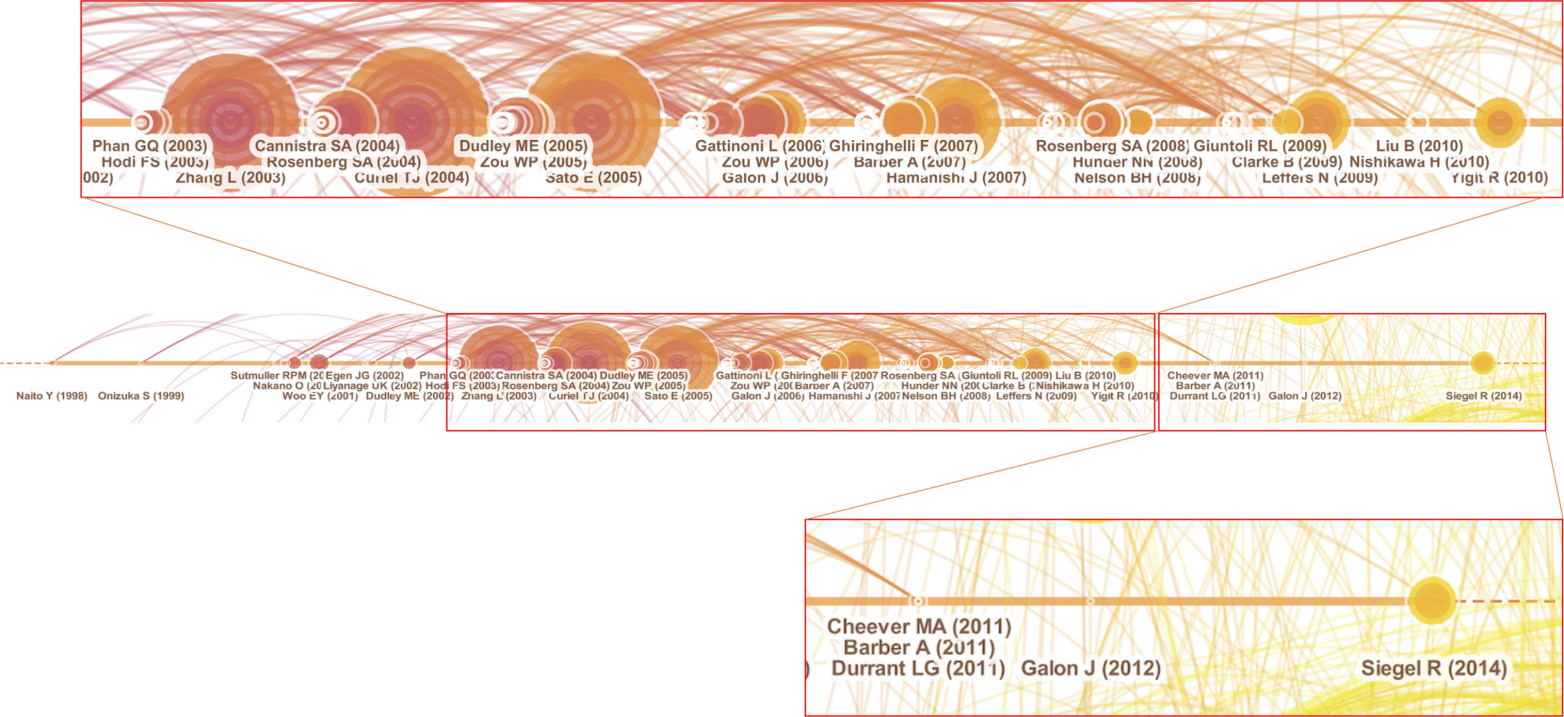
High impact members in cluster #1.

Cluster #1 contained 120 references with a silhouette score of 0.899, and the median year of all the references in the cluster was 2006. Natural killer group 2-member D (NKG2D) is a marker expressed on immune cells. NKG2D-based chimeric antigen receptor construct (chNKG2D) T cell belongs to adoptive T cell transfer and triggered a period of research burst. Timeline visualization revealed three periods of development for the cluster (**Figure 7**). The first period was from 1998 to 2002. This period was relatively uneventful and had few high-impact references in terms of citation numbers or bursts. One adoptive cell transfer article (Dudley ME, 2002) was followed by a second period of prevalence full of high-impact contributions in the field.

The second period was from 2003 to 2010. Contrary to the first period, many significant and highly cited publications were produced during this period. Below is a summary of publications that represent the main types of major contributions that appeared in this period:

Chimeric receptor – Chimeric NKG2D receptor T cells (Barber A, 2007).
Immune microenvironment – CTLA-4 (Phan GQ, 2003) (Hodi FS, 2003), PD-L1 (Hamanishi J, 2007), Regulatory T cells (Curiel TJ and 2004) (Sato E, 2005) (Zou WP, 2006) (Ghiringhelli F, 2007), and T cell infiltration (Zhang L and 2003) (Clarke B, 2009).
Cancer vaccine (Rosenberg SA, 2004).
Adoptive cell transfer (Dudley ME, 2005).

The third period was from 2011 to 2014. Although there was no burst of highly impactful publications during this period, some cited publications provided new insights into the specialty. The most cited publications in the third period included adoptively transferred chimeric NKG2D receptor-expressing T cells (Barber A, 2011) and an immune scoring system (Galon J, 2012). Barber et al. reported adoptive transfer of chimeric NKG2D receptor-expressing T cells to treat multiple myeloma and achieved promising tumor-free survival in mice (26). Galon et al. established an immune score based on lymphocyte infiltration and inflammation to classify cancers and to act as a predictive tool (27).

## 4 Discussion

Our study is a snapshot of the trends in OC immunotherapy research over the last 20 years. We gained several insights into the discipline, research trends, and the disease.

Using our search strategy, we found a total of 1,729 published articles in the field of immunotherapy for OC. The publication rate increased annually during the period studied. Of note, during the 2000s, the publication trends were somewhat stable and had a significant upward shift in early 2011. This incline may be a result of the development of immunotherapeutic agents for treating OC that opened new options for treatment along with undiscovered insights for research.

The analysis of the geographic distribution of publications revealed some interesting insights. USA published the most articles, which underscores its leading contribution to the subject. The number of clinical trials for OC immunotherapy in USA overwhelmingly exceeded that around the world. Notably, China’s article output has risen rapidly since 2015 and accounted for the second largest number of publications. This demonstrates the rapid development of China’s scientific research level in recent years. However, clinical trials in China are still few compared to those in USA. Besides, China’s average number of citations per article was the least among the top 10 countries, suggesting that high quality research is still lacking. Furthermore, we found that European countries contributed to the field of OC. The registries of European patients provided multiple significant insights into the epidemiology of OC over the last several decades (28, 29).

Clustering analysis provided additional insights and identified 14 major clusters through extraction of keywords from article abstracts. Of note, the major specialties shifted over the last two decades. This study also demonstrates the differed flourishing time of each cluster to provide a heuristic bibliometric finding. This kind of finding could be a useful tool for further research in the following two aspects:

### 4.1 Current Subtopics

First, the latest subtopics might be helpful in the decision-making for promising research directions. ‘PD-L1 expression,’ ‘tumor reactive til,’ and ‘parp inhibitor’ are the current prominent subtopics.

#### 4.1.1 PD-1/PD-L1 Axis

The immunosuppressive signal pathway of PD-1/PD-L1 plays an important role in the regulation of immune environment and tumor development. Binding of PD-L1 to PD-1 activates the immunosuppressive response, which causes exhaustion of effector T cells and immune escape of tumor cells (30). A previous study revealed that 90% of high-grade serous ovarian carcinomas express PD-L1, and a 30% positivity rate was achieved with a cut-off of 50% of stained cells (31). Landskron et al. revealed that PD-1-positive monocytes isolated from ascitic fluid and blood of OC patients were associated with poor prognosis (32). The aforementioned studies show that PD-1/PD-L1 is a promising target for OC treatment. Currently, several checkpoint inhibitors targeting PD-1/PD-L1 are applied in clinical treatment. The main checkpoint inhibitor agents against PD-1 are nivolumab and pembrolizumab, while agents against PD-L1 are atezolizumab, avelumab, and durvalumab. Here, we discuss the currently reported clinical trials of PD-1/PD-L1 inhibitors.

Pembrolizumab is an artificial monoclonal antibody against PD-1. Several clinical trials using pembrolizumab have been reported. Keynote 028 (NCT02054806) is a large phase IB clinical trial that included patients with different advanced solid tumors, and the results of the ovarian cohort have been reported (33). One of the inclusion criteria was positive PD-L1, defined as expression in ≥ 1% of cells in tumor nests or stroma determined immunohistochemically. Of the 26 enrolled patients, one patient achieved complete response (CR) and two patients achieved partial response (PR); the ORR was 11.5%. Drug-related adverse events occurred in 69.2% of the patients. Although the patient population was small, the ORR was unsatisfactory. Keynote 100 (NCT02674061) was a continuation of Keynote 028 with expansion of the patient population and introduction of PD-L1 stain score as a predictive biomarker (34). The results showed 8% ORR in all patients. Interestingly, higher PD-L1 expression was correlated with higher response. Deficient mismatch repair (dMMR) was noted in approximately 2–4% of all cancers, and tumors with dMMR are particularly susceptible to mutations in repetitive DNA sequences, resulting in high levels of microsatellite instability (MSI-H) and have unique phenotype in the immune microenvironment (35, 36). Keynote 158 was designed to use pembrolizumab in patients with advanced MSI-H/dMMR solid cancer. Fifteen patients with OC were enrolled, out of which 3 had CR and 2 had PR, with the ORR being 33.3%. From the above-mentioned Keynote trials, the development of PD-1 inhibitors is evident, a shift from simply focusing on the treatment effect to predicting the treatment efficacy at the protein or genetic level, which is consistent with the results of cluster #1. This can make immunotherapy more meaningful and help find a balance between checkpoint inhibitors and treatment-related adverse events (TRAEs).

Nivolumab is another human monoclonal antibody that specifically binds to PD-1. Many clinical trials using nivolumab to treat OC have been reported. UMIN000005714 was the first clinical trial on the effectiveness of nivolumab in patients with platinum-resistant OC, and this publication was also the most cited article in cluster #0 (25). The trial showed a 15% ORR, and a 45% disease control rate is promising for platinum-resistant OC that is poorly sensitive to traditional chemotherapy. A new therapeutic strategy combining nivolumab with checkpoint inhibitors or antiangiogenic agents was evaluated. In NCT02498600, ipilimumab plus nivolumab showed a superior response rate and a longer PFS compared with nivolumab alone for persistent or recurrent epithelial OC (37). In addition, NCT02873962 was a phase II trial that assessed the efficacy of combined nivolumab and bevacizumab for relapsed OC (38). Among 38 enrolled patients, 18 had platinum-resistant and 20 had platinum-sensitive disease. The ORR was 40.0% in platinum-sensitive and 16.7% in platinum-resistant participants. The results revealed an active response in platinum-sensitive patients, but the response in platinum-resistant patients was still low, and no benefit was observed compared to the previous trial UMIN000005714. Nivolumab was also evaluated in comparison with gemcitabine or pegylated liposomal doxorubucin (PLD) for the treatment of platinum-resistant OC, but no treatment benefit in terms of improved overall survival was observed (JapicCTI-153004) (39).

Atezolizumab is a humanized monoclonal anti-programmed death ligand-1 (PD-L1) antibody. The first phase I clinical trial to assess the safety of atezolizumab in OC reported the occurrence of low-grade TRAEs, and no grade ≥ 4 TRAEs were observed, representing acceptable side effects (NCT01375842) (40). A phase III trial investigating atezolizumab vs placebo associated with carboplatin/paclitaxel/bevacizumab for stage III/IV OC (NCT03038100) showed no additional benefit of atezolizumab in terms of progression-free survival and overall survival (41). Another trial on combined atezolizumab with bevacizumab for platinum-resistant OC achieved 15% ORR (NCT01633970) (42). Several trials on avelumab have recently been reported. JAVELIN OVARIAN 100 (NCT02718417) revealed no median progression-free survival benefit for OC patients treated with a combination therapy of avelumab and chemotherapy followed by maintenance, or chemotherapy followed by avelumab maintenance, compared with chemotherapy alone (43). A series phase 3 trial, JAVELIN OVARIAN 200 (NCT02580058), designed for platinum-resistant or platinum-refractory OC patients, investigated the effect of avelumab combined with PLD compared with avelumab or PLD alone. However, neither of the avelumab treatment strategies prolonged progression-free survival or overall survival compared to PLD (44). These results suggest that the use of avelumab for first-line treatment of platinum-resistant OC is not recommended. As for durvalumab, the combination was variegated with tumor vaccine (45), PARP inhibitors (46–48), and hypomethylating agents (49).

Taken together, checkpoint inhibitors targeting PD-1/PD-L1 axis are effective but not ideal for OC. Of note, patient selection for the trials is crucial. The Keynote series trial of pembrolizumab ensured precise inclusion. By stratifying patients according to PD-L1 expression or HRD status, the ORR was improved. With further exploration of tumor mechanisms, more potential biomarkers will emerge. For platinum-resistant/refractory OC, the response rate is low regardless of drug selection and combination strategy. Further investigations of the chemoresistance mechanism and microenvironment to find new therapeutic targets are warranted.

#### 4.1.2 Tumor Active Tumor-Infiltrating Lymphocytes

Besides chemotherapy and antibody target therapy, elimination of tumor cells is largely dependent on the immune system. Neoantigens generated by tumor cells impede normal immune system development and form a unique tumor microenvironment (TME) (50). Tumor-infiltrating lymphocytes (TILs) refer to T cells that infiltrate the TME to kill tumor cells. However, the tumor escape mechanism, through blockage of effector functions of infiltrating T cells by a broad spectrum of immunosuppressive agents in the TME, hinders immune-mediated elimination of tumors (51). Moreover, OC has a unique immunosuppressive microenvironment due to its intraperitoneal dissemination and omental metastasis. For example, adipose tissues can secrete many types of cytokines and molecular factors to recruit macrophages to attenuate T cell function (52, 53). Several research studies have investigated the correlation between TIL and prognosis of OC and identified several T cell predictive biomarkers, such as CD4, CD8, CD3, CD103, and PD-1 (54).

From 1990s, clinical trials of TIL therapy have been performed. Aoki et al. conducted the first TIL therapy trial for patients with advanced or recurrent OC and reported clinical response in 5/7 and 9/10 patients in TILs transfer group and TILs combined with chemotherapy group, respectively (55). Most of the clinical trials on TILs for OC treatment were performed in the 1990s, and no recent studies had been reported (56, 57). An unreported but complete trial (NCT03287674) designed adoptive T cell therapy with TIL infusion combined with nivolumab to treat metastatic OC. Briefly, patients were treated with one dose of ipilimumab 14 days before undergoing surgery to harvest tumor material for TIL production, followed by *in vitro* expansion and activation for around 4–6 weeks. nivolumab (3 mg/kg) was administered on day 2 before TIL infusion and every 2 weeks for a total of 4 doses. Twelve months after TIL and nivolumab combined therapy, six patients completed the trial (1 had PR and 5 had stable disease). No disease progression was observed. The main adverse events were neutropenia, thrombocytopenia, and anemia. The advantage of this combination therapy is that checkpoint inhibitors block the immunosuppression pathway and enhance TIL function. Despite the small sample size, this new combination strategy of checkpoint inhibitors and TIL is promising and should be verified in a larger population.

#### 4.1.3 PARP Inhibitor

Deficiency in the repair of DNA damage can result in genomic variation and increase the tumor mutational load (58). Besides their role in synthetic lethality, PARP inhibitors can lead to the production of neoantigens and DNA accumulation in the cytosol, which can trigger interferon pathways that enhance the immune response (59, 60). Jiao et al. reported that PARP inhibitors can upregulate PD-L1 expression *via* an interferon-independent pathway (61). Thus, combining PARP inhibitors and immunotherapy is a potential therapeutic strategy for OC. Several clinical trials on the combined therapy of PARP inhibitors and immune checkpoint blockade have been reported. The MEDIOLA trial is a phase ½ basket study that evaluated the combination of durvalumab (anti-PD-L1) and laparib in patients with platinum-sensitive relapsed OC (62). A total of 34 patients were enrolled, and the ORR was 63% (6 CR and 14 PR). In addition, PARP inhibitors combined with double immunotherapy [durvalumab and tremelimumab (CTLA-4)] for solid cancer were also evaluated (NCT04169841) (47).

Some concerns emerged from the current trials. First, the selection of PARP inhibitors in OC patients was determined by BRCA1/2 mutation or HRD status. Whether OC patients without gene mutation or low tumor mutational load are suitable for a combination therapy of PARP inhibitors and immunotherapy still requires further investigation. Moreover, the detection methods and diagnostic standards of HRD status should be unified. Second, PARP inhibitors are commonly used for maintenance therapy, and thus, more evidence is needed on the clinical benefit of the combination or sequential therapy and predictive biomarkers.

### 4.2 Subtopics With Breakthroughs

Notably, by adopting the newly developed ideas and technologies, breakthroughs could also be made in areas that flourished in the early periods. Although some subtopics are no longer prevalent, we can still illustrate new insights from their prior prevalence and their extinction process. For example, the subtopic ‘chnkg2d T cell’ was one of the biggest clusters. We can further understand from the change of high-impact references that researchers’ attention gradually shifted from the research of the treatment mechanism to the overall prognostic prediction of the patient outcomes. This coincided with a decrease in blind treatment. In recent years, more concepts and mechanisms about NKG2D have been explored. Soluble factors, such as TGF-β and IL-10, secreted by regulatory T cells (Tregs) and myeloid-derived suppressor cells (MDSC) from their microenvironment can downmodulate the expression of NKG2D-Ls (63). Moreover, NKG2D-ligands expressed in Tregs and MDSC suggests that CAR NKG2D cells could improve *in situ* immunosuppression (64). Thus, ch-NKG2D T cell therapy could target suppressive myeloid cells and enhance the function of subsequent infusions of tumor-specific CAR-T cells (65). These findings may precipitate a new research burst.

Oncolytic adenovirus was another subtopic with potential breakthroughs. Oncolytic adenoviruses, belong to a type of tumor vaccines, have advantage with large packaging capacity, lack of integration into the host genome and the mild side effect after infection making them attractive for tumor gene therapy (66, 67). Its specifically replicate within tumor cells, expressing target gene and greatly triggering the host’s immune response. Big Challenges for gene therapy in OC can conclude in following aspects: limitation of adenoviral infection and replication, physical barriers of massive ascites and immunosuppressive TME (68). Numbers of research have been reported to further overcome the obstacles for oncolytic adenovirus application in recent years. Due to properties of oncolytic adenovirus, combination strategies with other emerging targeted strategies, such as immune checkpoint inhibitors or CAR-T cells are expecting. Moreover, fully exploration of virus interaction with host is necessary.

Our study has some limitations. First, all the analyzed articles were extracted from WOSCC; therefore, not all published articles were included. Second, some of the less studied articles are not displayed through bibliometric algorithms, and thus relevant research findings may be omitted.

## 5 Conclusions

Our goal was to provide a reliable historiographic survey of the immunotherapy of OC. The survey identified the major clusters in terms of their high-impact publications and citation of articles that form new research fronts. We also discussed new insights that one can intuitively obtain through an inspection of citation trajectories and the positions of cited papers. Researchers can utilize these visual analytic tools to perform timely surveys of the literature as frequently as they wish and find relevant publications more effectively. Our interpretation not only identifies thematic milestones of OC immunotherapy research but also characterizes the developmental stages of the underlying specialties and the dynamic transitions from one specialty to another.

## Data Availability Statement

The original contributions presented in the study are included in the article/**Supplementary Material**. Further inquiries can be directed to the corresponding authors.

## Author Contributions

Conceptualization, TY and JZ. Methodology, GJ and TY. Software, GJ and JH. Validation, QW, FS, and FC. Data curation, GJ. Writing—original draft preparation, GJ. Writing—review and editing, TY. Visualization, JZ. Supervision, JZ. All authors have read and agreed to the published version of the manuscript.

## Conflict of Interest

The authors declare that the research was conducted in the absence of any commercial or financial relationships that could be construed as a potential conflict of interest.

## Publisher’s Note

All claims expressed in this article are solely those of the authors and do not necessarily represent those of their affiliated organizations, or those of the publisher, the editors and the reviewers. Any product that may be evaluated in this article, or claim that may be made by its manufacturer, is not guaranteed or endorsed by the publisher.

## Abbreviations

CR, complete response; dMMR, deficient mismatch repair; HRD, homologous recombination deficiency; MDSC, myeloid-derived suppressor cells; MSI-H, high levels of microsatellite instability; NKG2D, natural killer group 2-member D; OC, ovarian cancer; PARP, Poly (ADP-ribose) polymerase; PD-L1, programmed death ligand-1; PLD, pegylated liposomal doxorubucin; PR, partial response; TIL, tumor-infiltrating lymphocytes; TME, tumor microenvironment; TRAEs, treatment-related adverse events; Tregs, regulatory T cells; WOSCC, Web of Science Core Citation.
