# Supplementary material for: Evolution of Immunotherapy for Ovarian Cancer from a Bird’s-Eye Perspective: A Text-Mining Analysis of Publication Trends and Topics
Source: Front Oncol. 2022 Feb 24;12:795129. doi: 10.3389/fonc.2022.795129 (PMC8907843; doi:10.3389/fonc.2022.795129)
Supplement: Supplementary file 2 [file Table_1.docx]

Supplemental Table 1. Search strategy

| Strategy | Terms |
| --- | --- |
| A | ‘ovarian cancer’ OR ‘ovarian neoplasm’ OR ‘ovary cancer’ OR‘ovary neoplasm’ OR ‘cancer of ovary’ OR ‘ovarian cancers’ |
| B | ‘Immunotherapy’ OR ‘Immunotherapies’ OR ‘active immunotherapy’ OR ‘adoptive immunotherapy’ OR ‘passive immunization’ |
| C | A AND B |
